# Phylogenetic analysis linked fatal neurologic disease in leopards (*Panthera pardus*) to Asia-5 lineage of canine distemper virus in Nepal

**DOI:** 10.1016/j.virusres.2024.199463

**Published:** 2024-09-25

**Authors:** Amir Sadaula, Prajwol Manandhar, Bijaya Kumar Shrestha, Parbat Jung Thapa, Suresh Nepali, Janardan Dev Joshi, Babu Ram Lamichhane, Rachana Shah, Madhu Chetri, Kiran Raj Rijal, Kamal Prasad Gairhe, Naresh Subedi, Chiranjibi Prasad Pokheral, Roji Raut, Purushottam Pandey, Bikalpa Karki, Gita Pandey

**Affiliations:** a National Trust for Nature Conservation, Khumaltar, Lalitpur, Nepal; b Center for Molecular Dynamics Nepal, Thapathali, Kathmandu, Nepal; c Chitwan National Park Office, Kasara, Chitwan, Nepal; d Annapurna Conservation Area Office, Pokhara, Kaski, Nepal; e Kathmandu University, Dhulikhel, Kavre, Nepal; f Directorate of Livestock and Fisheries Development, Koshi Province, Biratnagar, Morang, Nepal; g Nepal Agricultural Research Council, Khumaltar, Lalitpur, Nepal

**Keywords:** Canine distemper virus, Leopards, *Panthera pardus*, Dogs, Spillover, Phylogenetic

## Abstract

Canine distemper virus (CDV) is responsible for a highly contagious and often fatal neurological disease that affects various carnivores, including domestic dogs. In Nepal, recent reports of CDV exposure and illness in leopards (*Panthera pardus*) have raised concerns about the transmission of the virus among domestic dogs and wild carnivores. To investigate the genetic lineage and spread of CDV, our study utilized archived post-mortem samples from four leopards that exhibited clinical signs suggestive of canine distemper infection. These leopards were rescued in the Palpa, Dolakha, Kathmandu, and Parbat districts. Our phylogenetic analysis revealed that the CDV strains circulating among the leopards belong to the Asia-5 lineage, which is also prevalent among dogs and wild carnivores in Nepal and neighboring India. The genetic relatedness between the leopard CDV sequences and those from both dogs and other carnivores within the Asia-5 lineage suggests that leopards in Nepal may have acquired the virus from multiple sources, potentially facilitated by their generalist dietary habits preying on dogs and even mesocarnivores. Furthermore, we inspected specific amino acid substitution in the hemagglutinin gene of leopard CDV, which also suggests possible transmission from both domestic dogs and non-canid hosts, although further research is needed to draw definitive conclusions. Given the vulnerable state of the leopard population in Nepal, already threatened by poaching and retaliatory killing, the emergence of CDV as a potential novel threat is deeply concerning. Comprehensive surveillance studies are essential to understand the dynamics of CDV spillover and to develop informed interventions. Urgent measures, including vaccination programs and effective control of the dog population, are needed to mitigate the impact of this disease and safeguard the future of Nepal's leopards and other wild carnivores.

## Highlights

• Canine distemper virus (CDV) poses a serious threat to leopards in Nepal, with recent raising concern of transmission in wildlife – domestic interface.
• CDV strains in leopards belong to the Asia-5 lineage, prevalent among dogs and wild carnivores in Nepal and neighboring India.
• Leopard in Kathmandu might have acquired CDV through dog predation, further research in wildlife in the region is required to draw definitive conclusions.
• Urgent measures, including vaccination for leopards and effective control of dog population, are recommended to minimize impact of CDV and safeguard leopard population.

## 1 Introduction

Canine distemper virus *Morbillivirus canis* (CDV)*,* a RNA virus of *Paramyxoviridae* family is highly contagious and often fatal to various carnivores, including domestic dogs, wild canids, large felids, and most Carnivora families (Loots et al., 2017). Its impact on threatened big cat species of the genus *Panthera* within their natural distribution range is evident, causing fatal outcomes (Wilkes, 2023). The disease spreads through direct aerosol contact, resulting in respiratory, gastrointestinal, and nervous symptoms with low chances of recovery (Zhao et al., 2015). A devastating outbreak in Tanzania in 1994, claimed the lives of over 1000 African lions (*Panthera leo leo*) and other carnivores in a month (Roelke-Parker et al., 1996). In 2018, an outbreak affecting the Asiatic lions (*Panthera leo persica*) in Gir National Park, India led to the death of over two dozen lions, and also affected other sympatric carnivores (Mourya et al., 2019). CDV has also caused fatalities in captive and wild Bengal tigers (*Panthera tigris*) and leopards (*Panthera pardus*) in India and, Nepal (Bodgener et al., 2023) and captive tigers and leopards in Europe and the USA (Appel et al., 1994; Kadam et al., 2022). In the Russian Far East, CDV has been isolated from endangered Amur tigers (*Panthera tigris altaica)* and critically endangered Amur leopards (*Panthera pardus orientalis*), further threatening their survival (Gilbert et al., 2020; Sulikhan et al., 2018).

In Nepal, CDV research has gained attention in recent years, especially considering the country's importance as a habitat for threatened wild carnivores such as Bengal tigers, leopards, snow leopards (*Panthera uncia*), dholes (*Cuon alpinus*), and wolves (*Canis lupus chanco*) (Adhikari et al., 2020). The country also has a substantial population of unmanaged free-roaming dogs (*Canis familiaris*), which could represent a potentially important reservoir of CDV and a source of infection for wild carnivores. Recent serosurveillance studies conducted on free−ranging dogs have demonstrated high levels of exposure to CDV (seroprevalence 17–80 %), confirming the circulation of the virus in several regions of Nepal (McDermott et al., 2023; Ng et al., 2019; Sadaula et al., 2022). The presence of CDV neutralizing antibodies in leopards and tigers in Nepal indicates exposure to the virus in the big cats as early as 2016 (Bodgener et al., 2023). This signifies that CDV transmission to wild carnivores has been occurring in the region.

In most outbreaks involving wild carnivores, dogs are often assumed to be the primary source of transmission or spillover (Cleaveland et al., 2000). However, the role of dogs is not inevitable and in some ecosystems, their role in the disease ecology may be relatively minor. For instance, in the Russian Far East, evidence suggests that abundant wild carnivores, including sable (*Martes zibellina*) play an important role as reservoirs and contribute to the infection of tigers and leopards (Gilbert et al., 2020). Similarly, in the case of African lions in Tanzania, it is suspected that CDV may be circulating among other wild carnivores, challenging the initial assumption that dogs were solely responsible (Nikolin et al., 2017; Viana et al., 2015). In Nepal, the higher seroprevalence in leopards (30 %, CI: 12.8–54.3 %, *n* = 20) compared to tigers (11 %, CI: 2.8–29.3 %, *n* = 28) suggests that dogs may be an important source of infection given their predominance in leopard diet compared to that of tigers (Bodgener et al., 2023). However, this remains speculative given the lack of sequencing data. In a recent study, it was found that CDV sequences from stray dogs in the Kathmandu Valley belong to the Asia-5 lineage, also found in dogs and wild carnivores in neighboring India (Bhatt et al., 2019; Kadam et al., 2022; Manandhar et al., 2023).

To investigate the potential transmission of CDV between dogs and wild carnivores in Nepal, we utilized archived post-mortem samples of leopards from various regions of Nepal that died exhibiting clinical signs consistent with canine distemper. Through RNA extraction and sequencing of CDV, we aimed to identify the virus lineage infecting leopards in Nepal, and relate it to published sequences of domestic dogs and other carnivores in the country and in South Asian region.

## 2 Materials and methods

### 2.1 Archived samples of leopards

The first leopard, an adult male leopard (Ppar1) was rescued from the Palpa district on 2016/05/18 (Table 1). The leopard was found in the middle of village and showed signs of dyspnea, open-mouth breathing and tonic-clonic seizures. The animal was immobilized by a response team from the National Trust for Nature Conservation (NTNC) and transported to Chitwan National Park (CNP) for further treatment. Despite intensive intravenous fluid therapy, the leopard died the following day with progressive seizures. The second leopard, an adult male (Ppar2), with tonic seizures was rescued by a team of the Division Forest Office, Dolakha on 2019/07/15 from a forest area. The animal developed progressive seizures and died the following day, despite treatment efforts by the team of Veterinarians. The third leopard was an adult male (Ppar3) that was wondering in the street of Kathmandu valley and it was rescued by the Division Forest Office, Kathmandu, and sent to the Central Zoo for further treatment. The animal displayed dyspnea and seizures and died after two days of treatment. The fourth leopard was an adult female leopard (Ppar4) and was found wandering in an agricultural field in Phalebas-2, Thapathana of Parbat district on 2023/03/03 and exhibited signs similar to those observed in leopard Ppar2. The NTNC response team was mobilized to rescue the leopard but it died prior to arrival (Supplementary Fig. 1).

**Table 1:** Case details and post-mortem tissue samples collected from leopards that died with clinical signs consistent with CDV.

Leopard ID	District	Date	Sex	Age	Tissue type
Ppar1	Palpa	2016/05/18	Male	Adult	Brain and Urinary bladder
Ppar2	Dolakha	2019/07/15	Male	Adult	Kidney
Ppar3	Kathmandu	2021/10/05	Male	Adult	Brain
Ppar4	Parbat	2023/03/03	Female	Adult	Brain and Urinary bladder

**Fig. 1:**
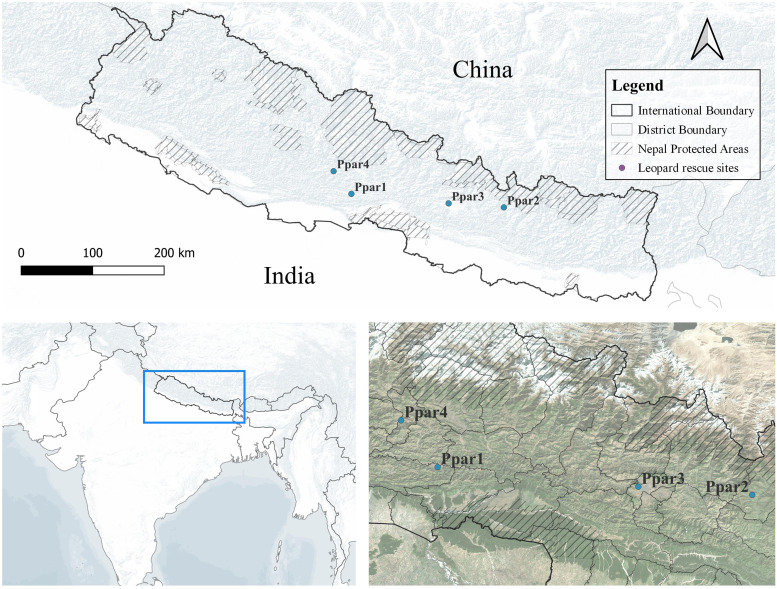
The geographical distributions of the leopards that exhibited clinical signs consistent with canine distemper in Nepal.

We carried out post-mortem examinations of the deceased leopards, and samples from the brain and/or urinary tissues (bladder or kidney) were collected and stored in a −20 °C freezer at NTNC laboratory for further investigations (Table 1, Fig. 1).

### 2.2 Molecular diagnosis and characterization of CDV in leopards

The post-mortem tissue samples were cut into small pieces and were vortexed in Trizol to form a homogenous suspension. We performed RNA extraction using the Quick-RNA Viral Kit-DX (Zymo Research, USA). We carried out cDNA synthesis from the RNA elute using Invitrogen Superscript III (ThermoFisher, USA). The prepared cDNA was quantified using Qubit dsDNA HS kit (ThermoFisher, USA). Both the RNA and cDNA were stored in a −20 °C freezer and 5x diluted cDNA were used for downstream processing.

To detect and characterize CDV, we amplified the partial *phosphoprotein (P)* gene (∼390 bp) and the full *hemagglutinin (H)* gene (approximately 1824 bp) of CDV via PCR using their respective primers (Barrett et al., 1993; Müller et al., 2011). The amplicons were visualized using 1.5 % agarose gel electrophoresis. The *P* gene was utilized as a general marker for confirmation, while the *H* gene served as the primary marker for further characterization of the CDV lineage, as previously described (Manandhar et al., 2023). The *H* gene encodes the viral surface glycoprotein that mediates attachment between the virus and the host cells. This gene has higher genetic variability than other portions of the genome and is commonly used as a marker for lineage classification of CDV (Bolt et al., 1997).

### 2.3 Sequencing of CDV phosphoprotein and hemagglutinin genes

PCR amplicons that showed expected bands on gel electrophoresis were sequenced using either Nanopore or Sanger sequencer. For Nanopore sequencing, the samples were purified using 0.8X Ampure beads and quantified with Qubit BR dsDNA Assay Kit. The quantified DNA was then normalized and processed for Nanopore library preparation. The normalized library was end-prepped using NEBNext Ultra II End Prep Reaction Mix, and native barcode ligation was performed using the Nanopore SQK.NBD112.24 kit and T4 ligase. Full-length *H* gene amplification was performed using a tiled amplicon method with four primer sets, and the resulting four amplicons from each sample were indexed with the same barcode. Among the six samples amplified for the *H* gene, 5 were barcoded, while 3 *P* gene samples (positive) were barcoded for Nanopore sequencing. The barcoded library of 8 samples was subsequently purified, normalized and pooled together for adapter ligation. The final library was further purified, quantified, and 15 femtomoles of the final pool were loaded onto a Flongle (FLO.FLG001) flowcell for sequencing in MinION (Oxford Nanopore Technologies, UK) device. The *H* gene amplicon from one remaining sample was sequenced using the ABI3730xl Genetic Analyzer (Applied Biosystems, USA) following the protocols outlined in Manandhar et al. (2023).

The raw sequences were analyzed using *NGSSpeciesID* pipeline for Nanopore based barcoding (Sahlin et al., 2021), and contig sequences for each *P* and *H* gene fragments were retrieved. The fragments were concatenated to generate a full-length consensus sequence of *H* gene for further analysis. For samples (sequences) originating from the same individual leopards, a single consensus sequence was used for subsequent analysis, given that they are identical.

The final processed sequences have been deposited in NCBI Genbank under accessions OR123688 to OR123692 and OR243301, and details are provided in Supplementary Table 1.

### 2.4 Phylogenetic analysis

To characterize the lineage of the CDV strains from the leopards, we compared the *H* gene sequences collected in our study with published sequences representing CDV lineages from India, Nepal and other regions of the globe. We compiled a dataset of 55 CDV *H* gene sequences (hereafter, referred as “reference sequences”) originating mainly from countries within South Asia, Russia, and Africa. This dataset includes vaccine strain (America-1) sequences along with major geographical lineages such as Asia-5, Africa-2, and Arctic-like that are known to have been previously sequenced from large felids along with other wild carnivores and dogs. All the data are publicly available in NCBI Genbank and are detailed in Supplementary Table 2.

We aligned all the reference sequences along with the leopard CDV sequences using MUSCLE ver3.8.425 (Edgar, 2004), and visually inspected alignment and then trimmed and edited the sequences wherever required using AliView ver1.26 (Larsson, 2014). For the phylogenetic tree reconstruction, we selected the best-fit nucleotide substitution model (TPM1uf+G) based on Bayesian Information Criterion in jModeltest2 ver2.1.8 (Darriba et al., 2012) from the alignment dataset. We conducted phylogenetic analysis using this model and the Bayesian inference method in MrBayes v3.2.7 (Ronquist et al., 2012) with 1,000,000 iterations, sampling every 2000 iterations and discarding the first 25 % as a burn-in. We visualized and annotated the phylogenetic tree using FigTree v1.4.4 (Rambaut, 2012).

The alignment of selected CDV-*H* amino acid sequences at receptor-binding site i.e. 501aa to 550aa were visually inspected, mainly for amino acid substitutions at site 549 of the four *H* protein sequences from the present study. This site is hypothesized to play a crucial role in CDV infectivity among carnivores (McCarthy et al., 2007). Specifically, CDV-*H* in canids typically has a tyrosine (Y) at this position, while a substitution to histidine (H) is believed to facilitate the virus's shift from dogs/canids to non-canid hosts (McCarthy et al., 2007).

## 3 Results

### 3.1 Molecular confirmation of fatal CDV infection in leopards

We tested four samples from two leopards (Ppar1 and Ppar4) for the *P* gene via PCR, and all four samples produced positive bands, although one sample (PG3) showed only a faint band. We successfully obtained *P* gene sequences from three samples (average depth at 2,226x), excluding the faint band sample (Supplementary Table 1). The sequences from PG1 and PG2 (Ppar4) were identical as expected, while they showed 97 % similarity to the PG4 sequence from Ppar1. BLAST analysis of PG1 revealed a 98 % similarity with the sequences: Vanguard vaccine strain (accession EU072201) and CDV dog strain in Thailand (accession JX886799). Similarly, PG4 showed 98 % similarity to sequences from CDV strains isolated from a dog in Bangladesh (accession OR880602), a jackal in India (accession MW876862), and the Vanguard vaccine strain (accession EU072201). The remaining samples from two leopards were directly sequenced for *H* gene. These results confirm the presence of CDV RNA in the post-mortem tissues of leopards that died with clinical signs consistent with CDV infection.

### 3.2 Phylogenetic characterization of CDV in leopards

**Fig. 2:**
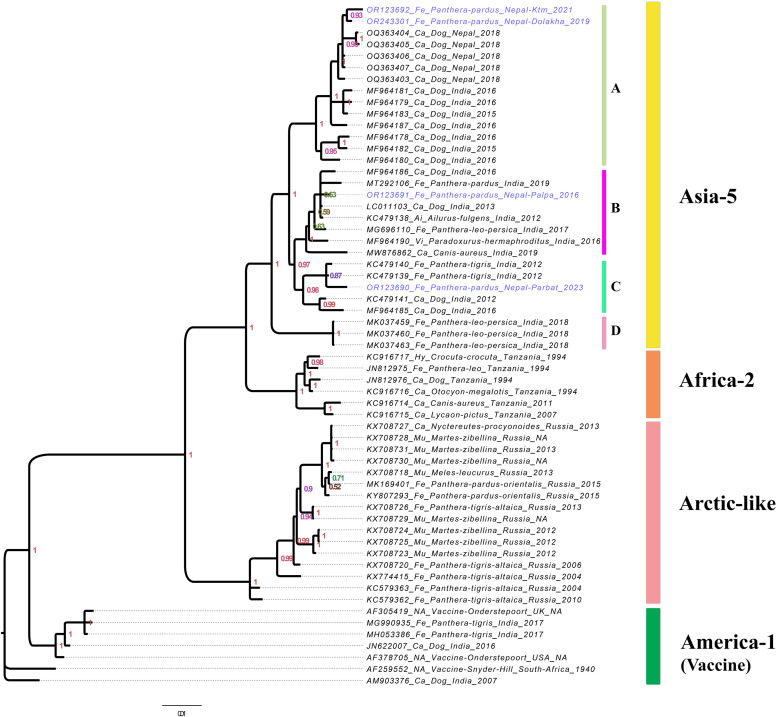
Phylogenetic tree reconstructed based on 1824 bp of CDV *H*-gene sequences from Asia-5, Africa-2, Arctic-like and America-1 lineages using Bayesian inference in MrBayes v3.2.7. The four sublineages of Asia-5 lineage are annotated as A, B, C and D. The four leopard CDV sequences from Nepal generated in this study are colored in blue. The values on the nodes are posterior probability estimated by the Bayesian inference method.

We obtained sequences of *H* gene from all five samples (average depth at 962x) sequenced through Nanopore and one sample (HG6) through Sanger sequencer (Supplementary Table 1). The *H* gene based phylogenetic analysis showed that the CDV sequences of leopards from Nepal belonged to Asia-5 lineage (Fig. 2), which has also been detected previously among dogs in the Kathmandu Valley, and both dogs and wild carnivores in neighboring India.

Within the Asia-5 lineage, at least four sub-clades were identified based on the *H* gene phylogeny, annotated as A, B, C and D in the Fig. 2. The four leopard CDV sequences clustered in three different subclades. The sublineage *A* consisted of strains found in dogs from Nepal's Kathmandu Valley and India's Uttar Pradesh. The CDV strains from two leopards from the Dolakha and Kathmandu districts clustered within this group. The sub-lineages *B* and *C* included strains from both dogs and wild carnivores from India. The CDV strains sequenced from two leopards from Palpa and Parbat districts in this study clustered within sublineages *B* and *C* respectively. The fourth sublineage *D* is the most distinct from others and consists solely of strains from Asiatic lions from Gir National Park, India.

**Fig. 3:**
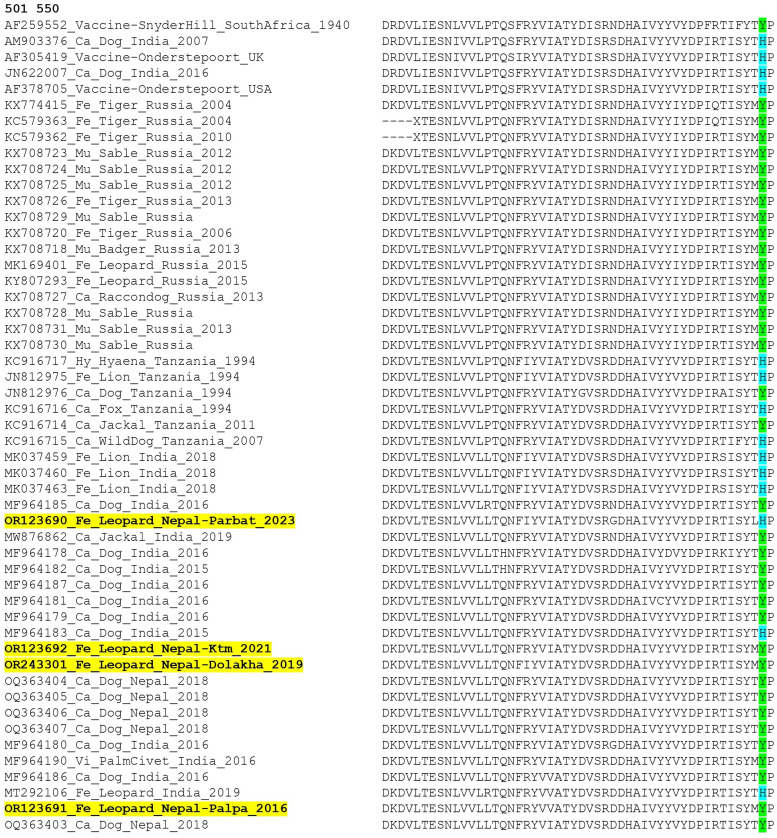
A sequence alignment of receptor-binding site (501aa to 550aa) of *Hemagglutinin* protein sequences of selected CDV-H protein, mainly highlighting substitutions at site 549 that is believed to be an important determinant of host infectivity of CDV according to McCarthy et al. (2007). Four leopard CDV sequences are highlighted in yellow. Amino acid residues highlighted in the position 549; green for Y and turquoise for H.

In the receptor-binding site of the CDV-*H* protein, the sequences from leopards in Palpa, Dolakha, and Kathmandu districts possessed amino acid Y at site 549 the Y549 residue, which is also present in dog CDV sequences from Kathmandu and India within sub-clade A (Fig. 3). The CDV sequence from Parbat district exhibited a Y549H substitution in the H protein (Fig. 3).

## 4 Discussion

Leopards in Nepal confront a multitude of threats, including conflicts, retaliatory killing, poaching, habitat loss, and declining prey populations. A recent study in Nepal has offered the initial confirmation of CDV exposure in leopards, suggesting that the disease may pose an additional threat to these big cats (Bodgener et al., 2023). The study revealed that leopards might be exposed to CDV more frequently than tigers, potentially due to their higher rates of dog predation, given that CDV is known to circulate among dogs in Nepal (Manandhar et al., 2023; McDermott et al., 2023; Ng et al., 2019; Sadaula et al., 2022). The study found CDV neutralizing antibodies in 30 % of leopards tested, while only 11 % of tigers showed evidence of exposure. Of the nine seropositive big cats, three individuals (one tiger and two leopards) showed clinical signs consistent with CDV and died shortly after sampling. Predation has been proposed as the most likely mode of inter-species transmission to big cats (Gilbert et al., 2020; Wang et al., 2022), which would be consistent with the higher exposure of leopards in Nepal considering the prominence of dogs in their diet (Bhattarai and Kindlmann, 2012; Kandel, 2019; Kandel et al., 2020; Koirala et al., 2012; Lovari et al., 2015). Genetic sequence data can aid in understanding the transmission pathways of viruses within and between species that is necessary to identify the source or reservoir of infection.

In previous studies, CDV strains obtained from dogs in Kathmandu Valley were identified as belonging to the Asia-5 lineage, which is also found in dogs and wild carnivores in neighboring India (Bhatt et al., 2019; Kadam et al., 2022; Manandhar et al., 2023; Mourya et al., 2019). Our phylogenetic analysis confirmed that the CDV strains obtained from all four leopards in our study also belonged to the Asia-5 lineage. However, each leopard's strain clustered in separate clades within the lineage (Fig. 2). The CDV strain from the Palpa leopard clustered in sublineage B, while the strains from the Dolakha and Kathmandu leopards clustered together in sublineage A. On the other hand, the strain from the Parbat leopard clustered in sublineage C. These sublineages, B and C, consisted of sequences from wild carnivores, including tigers and leopards, as well as a few dogs in India. This suggests that the Palpa and Parbat leopards may have acquired the virus through interactions with either wild hosts or possibly from dogs. Interestingly, the CDV sequences from the Dolakha and Kathmandu leopards clustered together with CDV sequences from dogs in Kathmandu within subclade A, which consists of only dog CDV sequences from Nepal and India. This finding suggests evidence of spillover between dogs and leopards, supporting previous speculations that dog predation may contribute to a higher CDV infection rate among leopards (Bodgener et al., 2023; Manandhar et al., 2023). Nevertheless, further research with sequences from other wild carnivores in the region is needed to draw definitive conclusions.

In our study, sequence analysis suggests that different dynamics may be influencing each of the recorded cases from four locations, which are approximately 40 to 180 km apart (Fig. 1). The amino acid residue at site 549 within the receptor-binding region of the CDV-*H* protein is speculated to be an important determinant of infectivity in carnivores (McCarthy et al., 2007). The substitution of tyrosine (Y) with histidine (H) at site 549 of the CDV-*H* receptor-binding site is thought to contribute to the spread of CDV from dogs to non-dog host species (McCarthy et al., 2007). Most,CDV strains isolated from Canidae have Y at site 549, whereas other carnivore families predominantly have H (Nikolin et al., 2012). In this study, the Y549H substitution was identified in the receptor-binding site of CDV-*H* from a leopard in Parbat, while all other leopards from Palpa, Dolakha and Kathmandu retained Y at site 549 (Fig. 3). Similarly, CDV sequences from dogs in Kathmandu (Manandhar et al., 2023) also possess Y549 (Fig. 3), consistent with dog sequences from India, and the majority of CDV strains from canids elsewhere (Nikolin et al., 2012). The impact of such amino acid substitutions on CDV transmission dynamics should be interpreted with caution, as non-dog hosts like mustelids and large felids sampled from Russia (Gilbert et al., 2020) have also been found to have Y549 (Fig. 3), which is typically associated with canid adaptation (Nikolin et al., 2012). More sequences from other carnivores, including mustelids, viverrids and canids are needed to clarify the transmission pathways. There could be several factors contributing to the spread of CDV, and various dynamics might be at play.

Since leopards are solitary felids, opportunities for intra-specific transmission are rare, and therefore infections require spillover from more abundant reservoir hosts (Wilkes, 2023). Free−ranging dogs could serve as potential reservoirs, especially considering their higher density in urban areas such as the Kathmandu Valley (Kakati, 2012). Nevertheless, sylvatic cycles involving wild reservoirs in South Asia may also play a vital role. This is suggested by the fact that several wild carnivores, including tigers, lions, leopards, and civets from protected areas, where dogs are not as densely present as in urban settings, have been found to be infected (Kadam et al., 2022). However, there is currently insufficient data to support this theory yet, unlike the evidence observed in the Russian Far East, where the abundance of small carnivores like sables has been linked to infection in Amur tigers and leopards (Gilbert et al., 2020). The CDV is known to infect a wide range of host species in urban, peri-urban and sylvatic environments. The domestic and wildlife interface plays a crucial role in maintaining the infection, with or without clinical signs (Duque-Valencia et al., 2019). In Nepal, both urban and sylvatic cycles may contribute to the maintenance and circulation of the virus in nature. In the wild, it is difficult to contain the virus within a species of solitary nature like large felids, and meta-reservoirs appear to sustain CDV. Wild carnivores such as martens, civets, mongooses and jackals are widespread in Nepal and are adaptable to human-dominated landscapes. Given that such settings are common throughout the country, mesocarnivores with invasive behavior and synanthropic habits may act as wild reservoirs (Reinhardt et al., 2023). On the other hand, free-ranging dogs potentially serving as urban reservoirs (Sadaula et al., 2022), collectively contributes to meta-reservoirs that help contain the virus in nature. In such a scenario, leopards may face an increased risk of exposure to CDV as they prefer diverse prey, as observed in various studies (Kandel et al., 2020; Koirala et al., 2012).

In general, leopard populations in Nepal and South Asia, already face significant challenges due to poaching and human-wildlife conflicts (Baral et al., 2022; Paudel et al., 2020; Rana and Kumar, 2023), and the risks posed by diseases like canine distemper may worsen the situation if the CDV prevalence reach unsustainable levels in the population. Notably, leopard populations have shown the ability to tolerate approximately 22 % adult mortality annually and still maintain their numbers (Chapron et al., 2008) However, CDV outbreaks have known to cause mass mortality in wildlife populations (Loots et al., 2017), potentially posing an unknown threat to leopards already facing anthropogenic pressures. Current data indicates a 30 % exposure rate to CDV in leopards, with only a few reported cases of neurological (fatal) outcomes in the prior study (Bodgener et al., 2023) and in the present study. Mortality in the wild often goes unrecorded, making the observation of at least four infected leopards in human spaces particularly significant. This is especially concerning given the sensitivity of small populations and the solitary nature of leopards, which make them vulnerable to stochastic events. (Gilbert et al., 2014). The solitary nature of leopards generally limits the circulation of infections, so spillover events generally involve multiple wild or domestic reservoirs (Sulikhan et al., 2018).

Despite our research sprinkling light on the CDV infection routes in leopards, there remains a significant knowledge gap regarding the dynamics of CDV spread among carnivores in Nepal and other regions. To enhance our understanding of CDV spillovers and reservoirs in the country, future surveillance efforts should encompass a broader range of species (Martinez-Gutierrez and Ruiz-Saenz, 2016), including dogs and various wild carnivores like jackals, martens, and civets (Kadam et al., 2022). Such research will provide valuable insights for wildlife management interventions, especially concerning the potential impact on threatened species like tigers, leopards, snow leopards, dholes and wolves (Adhikari et al., 2020). Direct wildlife surveillance is often impractical in resource-limited countries like Nepal. Therefore, any reported wildlife mortality cases must be evaluated, with samples archived for future retrospective studies. For instance, all our samples were archived from leopards rescued at various times, which later died with symptoms consistent with CDV infection. The availability of CDV sequences from dogs in Kathmandu Valley (Manandhar et al., 2023) suggests that the leopards rescued in Kathmandu and possibly Dolakha likely acquired the infection through dog predation, although this cannot be confirmed due to the lack of data from other wild carnivores in the region. Future research may be able to deduce the hypothesis with sequences from other wild carnivores. Thus, archiving such samples is crucial, as retrospective studies like ours can use them to investigate the potential etiology and epidemiology of diseases that lead to animal deaths.

Leopards, the largest predators in most of Nepal's mid-hill ecosystems, are potentially experiencing population declines from human-caused mortality (Baral et al., 2022; Paudel et al., 2020). The recent discovery of the risk of fatal CDV infection further highlights the need for future investigations to better understand the extent of this additional concern. More than 60 % of leopards’ range in Nepal lies outside protected areas, significantly increasing their vulnerability to disease exposure. By analyzing a broader range of CDV sequences, we can obtain further insights into the disease's ecology and epidemiology, enabling more informed decisions on interventions. Given the wide host range of CDV, the vaccination of domestic dogs, historical reservoirs of the virus, may not be sufficient to control the infection within a complex multi-host ecosystem (Cleaveland et al., 2000). While vaccinating wildlife at risk could be a solution, there are logistical, financial, and accessibility challenges making this approach impractical. The low-coverage vaccination of vulnerable populations like leopards, which is more feasible, has been shown to reduce the long-term risk of CDV infections in their populations (Wang et al., 2022). Nonetheless, the safety and effectiveness of vaccines in non-domestic species remain uncertain and may be controversial (Wilkes, 2023). As an alternative approach, implementing nationwide animal birth control strategies, such as neutering free−ranging dogs that may act as urban reservoirs, could improve the survival probability of leopard populations by reducing their exposure to such fatal diseases.

## Funding

The research received no external funding.

## Institutional review board statement

This study utilized archived specimens collected from rescued wild animals as part of routine wildlife rescue and management conducted by the Government of Nepal. No animals were specifically procured or immobilized for the sole purpose of collecting samples for this study.

## Informed consent statement

Not applicable.

## CRediT authorship contribution statement

**Amir Sadaula:** Writing – original draft, Resources, Methodology, Investigation, Funding acquisition, Conceptualization. **Prajwol Manandhar:** Writing – original draft, Formal analysis, Data curation, Conceptualization. **Bijaya Kumar Shrestha:** Supervision, Investigation, Conceptualization. **Parbat Jung Thapa:** Resources, Investigation. **Suresh Nepali:** Investigation. **Janardan Dev Joshi:** Formal analysis. **Babu Ram Lamichhane:** Writing – review & editing, Resources, Conceptualization. **Rachana Shah:** Resources. **Madhu Chetri:** Writing – review & editing. **Kiran Raj Rijal:** Methodology, Investigation. **Kamal Prasad Gairhe:** Methodology, Conceptualization. **Naresh Subedi:** Writing – review & editing, Project administration. **Chiranjibi Prasad Pokheral:** Writing – review & editing, Project administration. **Roji Raut:** Writing – original draft, Investigation. **Purushottam Pandey:** Resources. **Bikalpa Karki:** Methodology. **Gita Pandey:** Writing – original draft.

## Declaration of competing interest

The authors declare that they have no known competing financial interests or personal relationships that could have appeared to influence the work reported in this paper.

## Data Availability

All the data generated have been detailed in Supplementary materials.

## Appendix Supplementary materials

Image, application 1

The viral genetic sequences obtained in this study have been deposited in the NCBI GenBank database. Accession numbers for these sequences can be found in Supplementary Table S1. The accession numbers are OR123688- OR123692, OR243301. Image, application 2

## References

[bib0001] Adhikari R.B., Shrestha M., Puri G., Regmi G.R., Ghimire T.R. (2020). Canine distemper virus (CDV): an emerging threat to Nepal's wildlife. Appl. Sci. Technol. Ann..

[bib0002] Appel M.J., Yates R.A., Foley G.L., Bernstein J.J., Santinelli S., Spelman L.H., Miller L.D., Arp L.H., Anderson M., Barr M. (1994). Canine distemper epizootic in lions, tigers, and leopards in North America. J. Vet. Diagn. Investig..

[bib0003] Baral K., Bhandari S., Adhikari B., Kunwar R.M., Sharma H.P., Aryal A., Ji W. (2022). Anthropogenic mortality of large mammals and trends of conflict over two decades in Nepal. Ecol. Evol..

[bib0004] Barrett T., Visser I., Mamaev L., Goatley L., Van Bressem M.-F., Osterhaus A. (1993). Dolphin and porpoise morbilliviruses are genetically distinct from phocine distemper virus. Virology.

[bib0005] Bhatt M., Rajak K., Chakravarti S., Yadav A., Kumar A., Gupta V., Chander V., Mathesh K., Chandramohan S., Sharma A. (2019). Phylogenetic analysis of haemagglutinin gene deciphering a new genetically distinct lineage of canine distemper virus circulating among domestic dogs in India. Transbound. Emerg. Dis..

[bib0006] Bhattarai B.P., Kindlmann P. (2012). Interactions between Bengal tiger (*Panthera tigris*) and leopard (*Panthera pardus*): implications for their conservation. Biodivers. Conserv..

[bib0007] Bodgener J., Sadaula A., Thapa P.J., Shrestha B.K., Gairhe K.P., Subedi S., Rijal K.R., Pandey P., Joshi J.D., Kandel P. (2023). Canine distemper virus in tigers (*Panthera tigris*) and leopards (*P. pardus*) in Nepal. Pathogens.

[bib0008] Bolt G., Jensen T.D., Gottschalck E., Arctander P., Appel M.J., Buckland R., Blixenkrone M. (1997). Genetic diversity of the attachment (H) protein gene of current field isolates of canine distemper virus. J. Gen. Virol..

[bib0009] Chapron G., Miquelle D.G., Lambert A., Goodrich J.M., Legendre S., Clobert J. (2008). The impact on tigers of poaching versus prey depletion. J. Appl. Ecol..

[bib0010] Cleaveland S., Appel M., Chalmers W., Chillingworth C., Kaare M., Dye C. (2000). Serological and demographic evidence for domestic dogs as a source of canine distemper virus infection for Serengeti wildlife. Vet. Microbiol..

[bib0011] Darriba D., Taboada G.L., Doallo R., Posada D. (2012). jModelTest 2: more models, new heuristics and parallel computing. Nat. Methods.

[bib0012] Duque-Valencia J., Sarute N., Olarte-Castillo X.A., Ruíz-Sáenz J. (2019). Evolution and interspecies transmission of canine distemper virus—An outlook of the diverse evolutionary landscapes of a multi-host virus. Viruses.

[bib0013] Edgar R.C. (2004). MUSCLE: a multiple sequence alignment method with reduced time and space complexity. BMC Bioinform..

[bib0014] Gilbert M., Miquelle D.G., Goodrich J.M., Reeve R., Cleaveland S., Matthews L., Joly D.O. (2014). Estimating the potential impact of canine distemper virus on the Amur tiger population (*Panthera tigris altaica*) in Russia. PLoS One.

[bib0015] Gilbert M., Sulikhan N., Uphyrkina O., Goncharuk M., Kerley L., Castro E.H., Reeve R., Seimon T., McAloose D., Seryodkin I.V. (2020). Distemper, extinction, and vaccination of the Amur tiger. Proc. Natl. Acad. Sci..

[bib0016] Kadam R.G., Karikalan M., Siddappa C.M., Mahendran K., Srivastava G., Rajak K., Bhardwaj Y., Varshney R., War Z.A., Singh R. (2022). Molecular and pathological screening of canine distemper virus in Asiatic lions, tigers, leopards, snow leopards, clouded leopards, leopard cats, jungle cats, civet cats, fishing cat, and jaguar of different states, India. Infect., Genet. Evol..

[bib0017] Kakati, K., 2012. Street dog population survey, Kathmandu 2012. Final Report to WSPA, 5.

[bib0018] Kandel S. (2019). *Panthera pardus* fusca (Family: Felidae) diet composition from Lamjung, Nepal. Environ. Ecol. Res.

[bib0019] Kandel S.R., Lamichhane B.R., Subedi N. (2020). Leopard (*Panthera pardus*) density and diet in a forest corridor of Terai: implications for conservation and conflict management. Wildl. Res..

[bib0020] Koirala R.K., Aryal A., Amiot C., Adhikari B., Karmacharya D., Raubenheimer D. (2012). Genetic identification of carnivore scat: implication of dietary information for human–carnivore conflict in the Annapurna conservation area, Nepal. Zool. Ecol..

[bib0021] Larsson A. (2014). AliView: a fast and lightweight alignment viewer and editor for large datasets. Bioinformatics.

[bib0022] Loots A.K., Mitchell E., Dalton D.L., Kotzé A., Venter E.H. (2017). Advances in canine distemper virus pathogenesis research: a wildlife perspective. J. Gen. Virol..

[bib0023] Lovari S., Pokheral C.P., Jnawali S., Fusani L., Ferretti F. (2015). Coexistence of the tiger and the common leopard in a prey-rich area: the role of prey partitioning. J. Zool..

[bib0024] Manandhar P., Napit R., Pradhan S.M., Rajbhandari P.G., Moravek J.A., Joshi P.R., Shrestha R.D., Karmacharya D. (2023). Phylogenetic characterization of canine distemper virus from stray dogs in Kathmandu Valley. Virol. J..

[bib0025] Martinez-Gutierrez M., Ruiz-Saenz J. (2016). Diversity of susceptible hosts in canine distemper virus infection: a systematic review and data synthesis. BMC Vet. Res..

[bib0026] McCarthy A.J., Shaw M.-A., Goodman S.J. (2007). Pathogen evolution and disease emergence in carnivores. Proc. R. Soc. B.

[bib0027] McDermott I., Gilbert M., Shah M.K., Sadaula A., Anderson N.E. (2023). Seroprevalence of canine distemper virus (CDV) in the free-roaming dog (*Canis familiaris*) population surrounding Chitwan National Park, Nepal. PLoS One.

[bib0028] Mourya D.T., Yadav P.D., Mohandas S., Kadiwar R., Vala M., Saxena A.K., Shete-Aich A., Gupta N., Purushothama P., Sahay R.R. (2019). Canine distemper virus in Asiatic lions of Gujarat State, India. Emerg. Infect. Dis..

[bib0029] Müller A., Silva E., Santos N., Thompson G. (2011). Domestic dog origin of canine distemper virus in free-ranging wolves in Portugal as revealed by hemagglutinin gene characterization. J. Wildl. Dis..

[bib0030] Ng D., Carver S., Gotame M., Karmasharya D., Karmacharya D., Man Pradhan S., Narsingh Rana A., Johnson C.N. (2019). Canine distemper in Nepal's Annapurna conservation area—Implications of dog husbandry and human behaviour for wildlife disease. PLoS One.

[bib0031] Nikolin V.M., Olarte-Castillo X.A., Osterrieder N., Hofer H., Dubovi E., Mazzoni C.J., Brunner E., Goller K.V., Fyumagwa R.D., Moehlman P.D. (2017). Canine distemper virus in the Serengeti ecosystem: molecular adaptation to different carnivore species. Mol. Ecol..

[bib0032] Nikolin V.M., Wibbelt G., Michler F.-U.F., Wolf P., East M.L. (2012). Susceptibility of carnivore hosts to strains of canine distemper virus from distinct genetic lineages. Vet. Microbiol..

[bib0033] Paudel P.K., Acharya K.P., Baral H.S., Heinen J.T., Jnawali S.R. (2020). Trends, patterns, and networks of illicit wildlife trade in Nepal: a national synthesis. Conserv. Sci. Pract..

[bib0034] Rambaut, A., 2012. FigTree v1. 4.

[bib0035] Rana A.K., Kumar N. (2023). Current wildlife crime (Indian scenario): major challenges and prevention approaches. Biodivers. Conserv.

[bib0036] Reinhardt N.P., Köster J., Thomas A., Arnold J., Fux R., Straubinger R.K. (2023). Bacterial and viral pathogens with one health relevance in invasive raccoons (Procyon lotor, Linné 1758) in Southwest Germany. Pathogens.

[bib0037] Roelke-Parker M.E., Munson L., Packer C., Kock R., Cleaveland S., Carpenter M., O'Brien S.J., Pospischil A., Hofmann-Lehmann R., Lutz H. (1996). A canine distemper virus epidemic in Serengeti lions (*Panthera leo*). Nature.

[bib0038] Ronquist F., Teslenko M., Van Der Mark P., Ayres D.L., Darling A., Höhna S., Larget B., Liu L., Suchard M.A., Huelsenbeck J.P. (2012). MrBayes 3.2: efficient Bayesian phylogenetic inference and model choice across a large model space. Syst. Biol..

[bib0039] Sadaula, A., Joshi, J.D., Lamichhane, B.R., Gairhe, K.P., Subedi, N., Pokheral, C.P., Thapaliya, S., Pandey, G., Rijal, K.R., Pandey, P., 2022. Seroprevalence of Canine Distemper and Canine Parvovirus Among Domestic Dogs in Buffer Zone of Chitwan National Park, Nepal.

[bib0040] Sahlin K., Lim M.C., Prost S. (2021). NGSpeciesID: DNA barcode and amplicon consensus generation from long-read sequencing data. Ecol. Evol..

[bib0041] Sulikhan N.S., Gilbert M., Blidchenko E.Y., Naidenko S.V., Ivanchuk G.V., Gorpenchenko T.Y., Alshinetskiy M.V., Shevtsova E.I., Goodrich J.M., Lewis J.C. (2018). Canine distemper virus in a wild far eastern leopard (*Panthera pardus orientalis*). J. Wildl. Dis..

[bib0042] Viana M., Cleaveland S., Matthiopoulos J., Halliday J., Packer C., Craft M.E., Hampson K., Czupryna A., Dobson A.P., Dubovi E.J. (2015). Dynamics of a morbillivirus at the domestic–wildlife interface: canine distemper virus in domestic dogs and lions. Proc. Natl. Acad. Sci..

[bib0043] Wang D., Accatino F., Smith J.L., Wang T. (2022). Contributions of distemper control and habitat expansion to the Amur leopard viability. Commun. Biol..

[bib0044] Wilkes R.P. (2023). Canine distemper virus in endangered species: species jump, clinical variations, and vaccination. Pathogens.

[bib0045] Zhao J., Shi N., Sun Y., Martella V., Nikolin V., Zhu C., Zhang H., Hu B., Bai X., Yan X. (2015). Pathogenesis of canine distemper virus in experimentally infected raccoon dogs, foxes, and minks. Antivir. Res..

