# Supplementary material for: Phylogenetic analysis linked fatal neurologic disease in leopards (Panthera pardus) to Asia-5 lineage of canine distemper virus in Nepal
Source: Virus Res. 2024 Sep 25;350:199463. doi: 10.1016/j.virusres.2024.199463 (PMC11460512; doi:10.1016/j.virusres.2024.199463)
Supplement: Supplementary file 1 [file mmc1.docx]

**Supplementary Materials**

**Supplementary Table S1.** List of CDV gene sequences generated in this study along with their sample details and Genbank accession numbers.

| **SampleID** | **Tissue type** | **LeopardID** | **District** | **Collection date** | **Gene^#^** | **Genbank accession** | **Sequencing Depth (x)** |
| --- | --- | --- | --- | --- | --- | --- | --- |
| HG1 | Brain | Ppar4 | Parbat | 2023-03-03 | H | OR123690 | 638.8 |
| HG2 | Urinary bladder | Ppar4 | Parbat | 2023-03-03 | H | - | 528.5 |
| HG3 | Urinary bladder | Ppar1 | Palpa | 2016-05-18 | H | - | 529.4 |
| HG4 | Brain | Ppar1 | Palpa | 2016-05-18 | H | OR123691 | 436.0 |
| HG5 | Brain | Ppar3 | Kathmandu | 2021-04-22 | H | OR123692 | 2675.2 |
| HG6 | Kidney | Ppar2 | Dolakha | 2019-07-15 | H | OR243301 | NA* |
| PG1 | Brain | Ppar4 | Parbat | 2023-03-03 | P | OR123688 | 2077.0 |
| PG2 | Urinary bladder | Ppar4 | Parbat | 2023-03-03 | P | - | 2574.5 |
| PG4 | Brain | Ppar1 | Palpa | 2016-05-18 | P | OR123689 | 2025.0 |

Gene^#^: H is hemagglutinin gene; P is phosphoprotein gene

NA*: Not applicable, as this sample is sequenced using a Sanger sequencer.

**
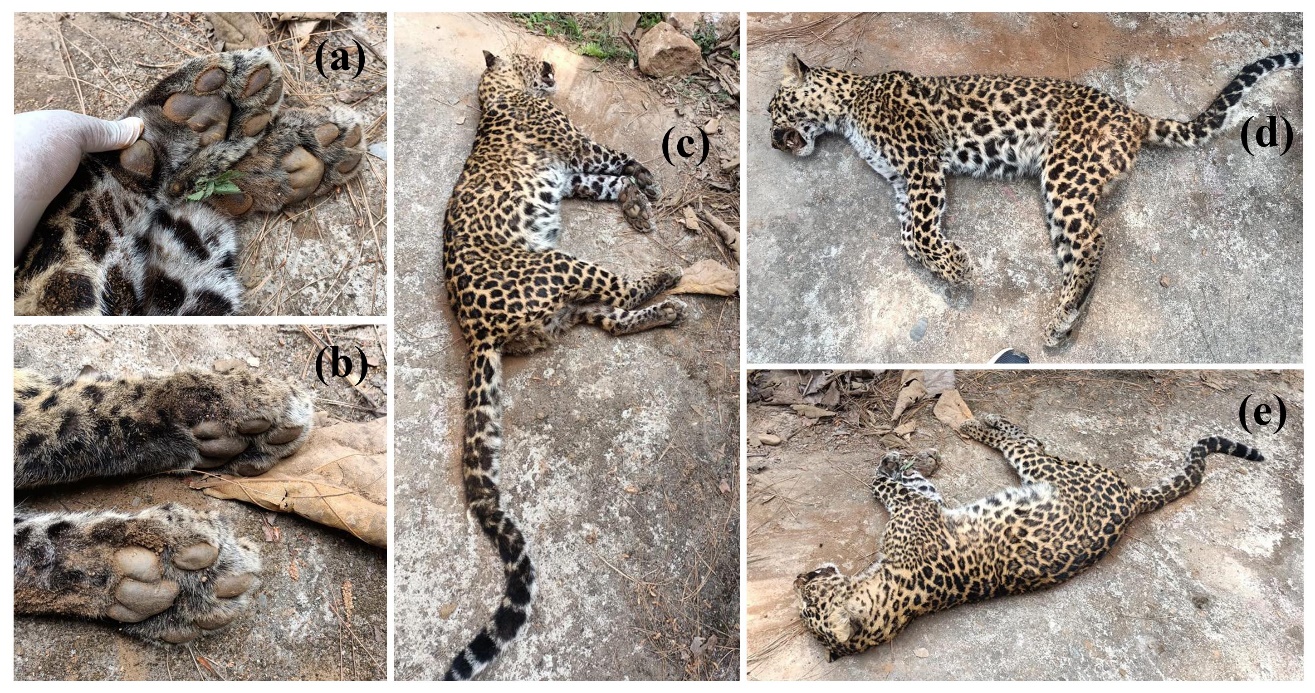
**

**Supplementary Figure S1.** The condition of female leopard ‘Ppar4’ in Parbat district on 2023/03/03. Later she died with clinical signs of CDV infection. Hyperkeratosis was observed on both fronts (a) and hind (b) footpads, which is a common symptom seen in CDV infected dogs. The body condition (c, d and e) appears to be emaciated and very poor.
